# The Development and Characterization of an Andiroba Oil-Based Nanoemulsion (*Carapa guianensis*, Aubl.): Insights into Its Physico-Chemical Features and In Vitro Potential Healing Effects

**DOI:** 10.3390/pharmaceutics17040498

**Published:** 2025-04-09

**Authors:** Isolda de Sousa Monteiro, Aimê Stefany Alves Fonseca, Carolina Ramos dos Santos, João Paulo Santos de Carvalho, Sebastião William da Silva, Valdir F. Veiga-Junior, Rayssa Ribeiro, Ivo José Curcino Vieira, Thalya Soares Ribeiro Nogueira, Carlos Alexandre Rocha da Costa, Gilson Gustavo Lucinda Machado, Lorrane Ribeiro Souza, Eduardo Valério Barros Vilas Boas, Samuel Silva Morais, Jackson Roberto Guedes da Silva Almeida, Livia Macedo Dutra, Victória Laysna dos Anjos Santos, Atailson Oliveira Silva, Marcelo Henrique Sousa, Marcella Lemos Brettas Carneiro, Graziella Anselmo Joanitti

**Affiliations:** 1 Laboratory of Bioactive Compounds and Nanobiotechnology (LCBNano), University of Brasilia, Campus Universitário—Centro Metropolitano, Ceilandia Sul, Brasília 72220-275, DF, Brazil; isoldamonteiro31@gmail.com (I.d.S.M.); aimefonseca@gmail.com (A.S.A.F.); crsbiotec@hotmail.com (C.R.d.S.); moraisssamuel@gmail.com (S.S.M.); marbretas@unb.br (M.L.B.C.); 2 Post-Graduation Program in Sciences and Technologies in Health, Faculty of Ceilandia, University of Brasilia, Campus Universitário—Centro Metropolitano, Ceilandia Sul, Brasília 72220-275, DF, Brazil; atailson.silva@unb.br (A.O.S.); mhsousa@unb.br (M.H.S.); 3 Laboratory of Optical Espectroscopy, Physics Institute, University of Brasilia, Campus Universitário Darcy Ribeiro, Brasília 70910-900, DF, Brazil; joaopaulocarv96@gmail.com (J.P.S.d.C.); swsilvaunb@gmail.com (S.W.d.S.); 4 Chemistry Section, Military Institute of Engineering, Praça Gen. Tibúrcio, 80, Praia Vermelha, Rio de Janeiro 22290-270, RJ, Brazil; valdir.veiga@ime.eb.br (V.F.V.-J.); rayssaribeiro_92@hotmail.com (R.R.); 5 Laboratório de Ciências Químicas-LCQUI, Universidade Estadual do Norte Fluminense Darcy Ribeiro-UENF, Avenida Alberto Lamego 2000, Campos dos Goytacazes 28013-602, RJ, Brazil; curcino@uenf.br (I.J.C.V.); thalyasrnogueira@gmail.com (T.S.R.N.); 6 Food Science Department—DCA, Federal University of Lavras—UFLA, Lavras CEP 37200-900, MG, Brazil; alexandre.vitae@gmail.com (C.A.R.d.C.); gilsonguluma@gmail.com (G.G.L.M.); lollaribeiros@gmail.com (L.R.S.); evbvboas@ufla.br (E.V.B.V.B.); 7 Núcleo de Estudos e Pesquisas de Plantas Medicinais (NEPLAME), Department of Pharmacy, Universidade Federal do Vale do São Francisco (UNIVASF), Petrolina 56304-917, PE, Brazil; jackson.rgsa@gmail.com (J.R.G.d.S.A.); liviamdutra@gmail.com (L.M.D.); victoria.laysna@univasf.edu.br (V.L.d.A.S.); 8 Green Nanotechnology Group, University of Brasilia, Campus Universitário—Centro Metropolitano, Ceilandia Sul, Brasília 72220-275, DF, Brazil

**Keywords:** *Carapa guianensis*, fixed oil, nanostructure, cell survival, cell migration assay

## Abstract

**Background/Objectives:** Andiroba oil, extracted from *Carapa guianensis* seeds, possesses therapeutic properties including anti-inflammatory and wound healing effects. This study aimed to develop and characterize a nanoemulsion formulation containing andiroba oil (NeAnd) and to evaluate its cytotoxicity and wound healing potential in vitro. **Methods**: The oil was evaluated for acidity, antioxidant activity, and fatty acid composition. NeAnd was produced by ultrasonication and characterized using FTIR (Fourier transform infrared spectroscopy), Raman spectroscopy, dynamic light scattering, and transmission electron microscopy. **Results:** NeAnd exhibited a spherical shape and stable physicochemical properties, with an average hydrodynamic diameter (HD) of 205.7 ± 3.9 nm, a polydispersity index (PdI) of 0.295 ± 0.05, a negative zeta potential of −4.16 ± 0.414 mV, and pH of approximately 6.5. These nanodroplets remained stable for 120 days when stored at 4 °C and maintained their parameters even under pH variations. FTIR and Raman analyses confirmed the presence of functional groups and the organization of fatty acid chains in NeAnd. Cell viability assays revealed no statistically significant differences in cytotoxicity at various concentrations (90–360 µg/mL) after 24 and 48 h. In scratch wound healing assays, NeAnd significantly enhanced wound closure (88.9%) compared to the PBS control (38%) and free andiroba oil (68.6%) in keratinocytes (*p* < 0.05). **Conclusions:** These promising findings indicate NeAnd as a potential nanophytomedicine for wound healing and tissue regeneration treatments.

## 1. Introduction

Wound healing involves cellular, biochemical, and molecular mechanisms such as fibroblast proliferation, collagen deposition, and the development of new blood vessels [1]. Non-healing wounds (particularly chronic wounds) result from disruptions in the healing process due to comorbidities (e.g., diabetes, immunosuppression), infections, and increased inflammation. Moreover, poor wound healing adversely affects the global economy and public health [2,3]. Several wound healing strategies have been employed for the treatment of chronic wounds [2,3]. However, given the complexity of wound types and their impact on public health, there is a growing need to develop new technologies aimed at improving healing quality and reducing treatment time [1,2,3].

Nanotherapies have rapidly gained attention in the field of wound healing, with various studies and techniques analyzing how nanoscale strategies can improve different phases of the wound repair process by enhancing effectiveness, bioavailability, and cellular interactions of therapeutic compounds [4,5,6,7,8,9]. Different types of nanostructures can be used as carriers for bioactive compounds involved in wound repair, such as nanoemulsions [4,5]. Nanoemulsions are heterogeneous systems stabilized by surfactants in which a liquid (dispersed phase) is dispersed within another immiscible liquid (continuous phase) as nanosized droplets [6]. The use of natural compounds with wound healing properties, such as plant fixed oils, has been described as a promising alternative for tissue regeneration [1,2,3]. Interestingly, their nanoencapsulation has emerged as a promising strategy to enhance their efficacy, stability, and safety [6,7,8,9,10].

Andiroba (*Carapa guianensis*, Aubl.) is a large tree predominantly found in Latin America, Africa, and the southern regions of the Sahara [11,12,13], from which a fixed oil with antiparasitic, antifungal, bactericidal, anti-inflammatory, and wound healing properties can be extracted [14]. Andiroba fixed oil (OA) is widely known and used in Brazilian folk medicine, particularly by the inhabitants of the Amazon rainforest, and its composition has attracted the interest of researchers seeking to understand its benefits and confirm its effectiveness [11,12,13,14]. In vivo studies have shown reduced healing times in OA-treated wounds [15,16,17]. The fixed oil from *Carapa guianensis* appears to decrease the density of collagen type I fiber networks, forming them in a reticular pattern and, notably, increasing the presence of type III fibers. Additionally, it promotes fibroblast proliferation and stimulates angiogenesis in the injury region, which is essential for tissue regeneration [12,18]. Nevertheless, to the best of our knowledge, no study has investigated whether nanostructuring OA can further enhance its wound healing effects. In addition to improved wound healing outcomes, nanotechnology can increase its therapeutic potential while promoting the responsible use of biological resources, benefiting both society and the environment [19,20].

Therefore, considering that nanotechnology could enhance OA’s wound healing properties, this study aimed to develop and characterize a nanoemulsion formulation based on andiroba fixed oil (NeAnd) and to investigate its cytotoxicity and wound healing potential in vitro. By demonstrating that NeAnd is biocompatible and capable of significantly accelerating keratinocyte migration and wound closure, this study provides new insights into its potential application as a nanophytotherapeutic agent for wound healing and tissue regeneration.

## 2. Material and Methods

### 2.1. Materials

The andiroba fixed oil was commercially acquired by the company FERQUIMA Ltda. (São Paulo, Brazil). The oil was extracted through cold pressing. Egg lecithin was purchased from Lipoid (Ludwigshafen, Germany). Hexane (P.A.) was acquired from Dinâmica Química Contemporânea LTDA (Indaiatuba, SP, Brazil). 3-[4,5-dimethylthiazol-2-yl]-2,5-diphenyltetrazolium bromide (MTT), methanol, and dimethyl sulfoxide (DMSO) were purchased from Sigma Aldrich Chemical Co. (St. Louis, MO, USA). Dulbecco’s modified Eagle’s medium (DMEM), fetal bovine serum (FBS), trypsin, penicillin, and streptomycin were all purchased from Thermo Fisher Scientific (Waltham, MA, USA).

### 2.2. Andiroba Fixed Oil Characterization

#### 2.2.1. Gas Chromatography–Mass Spectrometry (GC-MS)

The lipid profile was characterized according to the methodology described in our previous study [17]. To investigate the presence of other bioactive compounds in the oil, analyses by gas chromatography–mass spectrometer (GC-MS) were performed in an Agilent equipment (model 5975C masses coupled to a 7890 A gas chromatograph). Sample insertion was performed using a 10 µL syringe in a 7693A automatic injector. A capillary column (HP5 ms, 30 m × 250 µm × 0.25 µm of (5% phenyl)-methylpolysiloxane film) was used with helium as a carrier gas at a constant flow of 1.0 mL/min. Temperature was set at 50 °C for 1 min, rising to 180 °C (at 2 °C/min), followed by a rise to 250 °C (at 10 °C/min) for 10 min and finally rising to 280 °C. Mass spectrometric detection was performed, using the following parameters: interface, ionization source and quadrupole analyzer temperature at 280, 230, and 150 °C, respectively, and electron impact ionization (EI) mode at 70 eV. The results were identified by comparison with the equipment’s NIST library database, based on the similarity index and visual inspection of the mass spectra obtained versus the equipment’s library.

#### 2.2.2. Nuclear Magnetic Resonance (NMR) Analysis

The chemical characterization of andiroba oil was performed by nuclear magnetic resonance (NMR) analysis. For this, one-dimensional (1D) NMR data were acquired at 298 K in CDCl_3_ on a Bruker AVANCE III 400 NMR spectrometer (Bruker BioSpin GmbH, Rheinstetten, Germany) operating at 9.4 T, observing ^1^H and ^13^C at 400 and 100 MHz, respectively. The NMR spectrometer was equipped with a 5 mm multinuclear direct detection probe with a z-gradient. ^1^H NMR acquisition was acquired using zg30, with relaxation delay (D1) of 2.0 s, 64 k numbers of data (TD) over an 8012.8 Hz spectral width (SW) averaged over 8 numbers of scans (NSs), radiofrequency pulse (P1) of 14.28, acquisition time (AQ) of 4.08 s, dummy scans (DSs) of 2, and receiver gain (RG) of 4.5. Already, ^13^C NMR spectrum was acquired with the following acquisition parameters: pulse sequence (zgpg30), 32 k of TD, SW of 29761.9 Hz, 250 of NSs, P1 of 9.5 μs, AQ of 0.55 s, D1 of 0.50 s, 4 of DSs, and RG of 203. Then, ^1^H NMR spectrum was apodized via exponential Lorentzian broadening multiplication corresponding to 0.3 Hz line broadening (LB) in the transformed spectrum, while ^13^C NMR spectrum was apodized with LB of 1.00 Hz. ^1^H and ^13^C NMR chemical shifts (δ) were given in ppm related to the tetramethylsilane TMS signal at 0.00 as an internal reference, and the coupling constants (J) in Hz. The spectra were processed in the TOPSPIN software (https://www.bruker.com/en/products-and-solutions/mr/nmr-software/topspin.html, accessed on 30 March 2025).

#### 2.2.3. Peroxide and Acidity Index

Acid and peroxide values and free fatty acids were measured following the American Oil Chemists’ Society (AOCS) methods (Cd 3d-63, Cd 8b-90 and Ca 5a-40, respectively) and expressed as g KOH/g, meq O_2_/Kg, and % oleic acid, respectively (AOCS 2004) [21].

#### 2.2.4. Methods for Determining Antioxidant Activity

Two methods were used to determine antioxidant activity. The first method used was the Phosphomolybdenum Complex previously described [22], with results expressed in milligrams of ascorbic acid in 100 g of sample. The second method is based on the scavenging of the stable free radical 1,1-diphenyl-2-picrylhydrazyl (DPPH•), previously described [23]. The discoloration degree of 3.9 mL DPPH• methanolic solution 0.06 mM promoted by 0.1 mL of andiroba oil acetonic extract (0.5 g oil + 20 mL acetone) was measured in spectrophotometer, after one hour of reaction, in accordance with the equation:

% DPPH• discoloration = [(Abs DPPH• − Abs sample + DPPH•)/Abs DPPH•] 100

Reading was performed using a microplate reader (EZ Read 2000, Biochrom, Cambridge, UK) at 695 nm for phosphomolybdenum complex and 515 nm for DPPH•. Values were expressed as mean ± standard deviation. The analysis was performed in three repetitions.

#### 2.2.5. Total Phenolic Content and Total Carotenoids

The total phenolic content (TPC) was determined by the Fast Blue method described [24]. The results were expressed in milligrams of gallic acid equivalent (GAE) in 100 g of sample. Total carotenoids were determined using the spectrophotometric method previously described [25].

### 2.3. Development of Andiroba Fixed Oil-Based Nanoemulsion (NeAnd)

The development of the andiroba oil-based nanoemulsion (NeAnd) was an adaptation of a method previously described in Ombredane et al., 2020 [17]. Briefly, a coarse emulsion was prepared by adding egg lecithin and andiroba oil in PBS in the concentration of 32.4 mg of OA, and 68.4 mg of egg lecithin in 15 mL of PBS was prepared and then sonicated at 20 kHz, under an ice bath for 3 min. The formulations were stored at 4 °C in the dark until further analysis. A blank formulation (without the oil) was prepared similarly as described above.

### 2.4. Physicochemical Characterization of NeAnd

#### 2.4.1. Colloidal Characteristics

To evaluate the colloidal characteristics, hydrodynamic size followed by the polydispersity index and zeta potential were determined using ZetaSizer^®^ Nano ZS90 (Malvern, UK) through dynamic light scattering (DLS) and electrophoretic light scattering (ELS), respectively. The experiments were carried out in triplicate, 24 h after the preparation of the nanoemulsion samples, and by using Malvern-DTS0012 (DLS) and Malvern-DTS1070 (ELS) cuvettes. Before the analysis, samples were diluted (1:10, *v*/*v*) with distilled water to be optically clear and avoid multiple scattering and viscosity effects [18].

#### 2.4.2. Colloidal Stability of NeAnd over Time and at Different pHs

To evaluate the effect of storage time, NeAnd was maintained at 4 °C and protected from light, and their hydrodynamic size, polydispersity index, and zeta potential were analyzed after 1, 7, 15, 60, 90, and 120 days with the methods and equipment described in Section 2.4.1. The influence of pH on nanoemulsion stability was investigated by measurements of hydrodynamic size, polydispersion index and zeta potential at different pHs. The pH of the medium was modified using an automatic MPT-2 autotitrator (Malvern Instruments) with NaOH and/or HNO_3_ standard solution as titrants.

#### 2.4.3. Morphology of NeAnd by Transmission Electron Microscopy (TEM)

To analyze the shape/morphology of NeAnd, a drop of approximately 20 microliters of the sample was placed onto a piece of Parafilm, and a grid was placed over the drop for 10 min. Subsequently, a drop of 2% uranyl acetate solution (contrast) was added to the Parafilm, and the grid was placed over this drop for 1 min. The samples were air-dried at room temperature. After drying, the samples were analyzed using the Transmission Electron Microscope (TEM), JEM-2100, Jeol, Tokyo, Japan, operating at 200 kV.

#### 2.4.4. Infrared Spectrophotometry (FTIR) Analysis of NeAnd

To better elucidate the chemical interactions among NeAnd components, FTIR measurements were conducted using a Bruker Fourier transform infrared spectrometer, model Vertex 70. The analysis was performed using the attenuated total reflection module (ATR-FTIR). An average of 96 scans were collected for each sample, with a resolution of 4 cm^−1^, in the range of 400 to 4000 cm^−1^, with the same background sampling before each measurement. It is important to mention that the instrument’s wavenumber precision is better than 0.01 cm^−1^ at 2000 cm^−1^ in the ATR-FTIR measurement (Bruker Vertex 70). Therefore, wavenumber shifts of approximately 1 cm^−1^ are considered significant.

#### 2.4.5. Raman Analysis of NeAnd

To complement data obtained from FTIR measurements and better understand the structure organization of NeAnd components, RAMAN measurements were performed using the LabRAM HR Evolution spectrometer, manufactured by Horiba. The Raman spectrometer is equipped with a confocal microscope, a CCD (Charge Coupled Device) detector, and an 1800 lines/mm grating. The measurements of each sample were conducted using a He-Ne laser tuned to the 633 nm line (1 mW) and focused on the sample using a 50× objective lens.

### 2.5. Cell Culture

HaCat cells (human keratinocytes) were obtained from Cell Bank of Rio de Janeiro (Brazil) and cultured in Dulbecco’s modified Eagle’s medium (DMEM), supplemented with 10% (*v*/*v*) fetal bovine serum (*v*/*v*) and 1% of antibiotic solution (100 IU/mL of penicillin—100 µg/mL of streptomycin—*v*/*v*) at 37 °C and 5% CO_2_.

#### 2.5.1. Cytotoxicity Assay

To elucidate the effects of NeAnd and free andiroba oil in cell viability, cells were seeded into 96-well culture plates at a density of 5 × 10^3^ cells/well overnight at 37 °C, 5% CO_2_ in a humid atmosphere. Then, various concentrations of NeAnd, blank nanoemulsion (without andiroba oil), and free andiroba oil were added (90, 180, and 360 μg/mL, considering andiroba oil concentration). Due to its hydrophobicity, the free andiroba oil was previously dispersed in ethanol (EtOH) with final concentration of ethanol lower than 1% per well, which is nontoxic for cells. Corresponding cell control groups containing only ethanol (1% per well; for free OA) or PBS (for NeAnd and blank nanoemulsion) were used for data analysis. The plates were incubated for 24 and 48 h at 37 °C, 5%. The cell viability assay was performed using the MTT assay [26].

#### 2.5.2. Scratch Assay

To investigate the effects of NeAnd and free andiroba oil in cell migration, 8 × 10^4^ cells/well were seeded in 24-well plates and incubated overnight at 37 °C, 5% CO_2_ in a humid atmosphere. Then, the scratch assay was performed based on the protocol by Liakopoulou et al. (2021) [27]. After establishing the scratch line, the cells were exposed to 360 μg/mL of NeAnd, blank, oil, and controls for 24 h. For the treatment with free oil, a solution in EtOH was prepared with final concentration of ethanol lower than 1% per well. The scratch was recorded under an optical microscope before applying the treatment (T0) and after 24 h (T24). The migration records of HaCat cells were evaluated using ImageJ software (https://imagej.net/ij/, accessed on 30 March 2025) and the plugin described by Suarez-Arnedo et al. [28].

### 2.6. Statistical Analyses

Statistical differences between experimental groups were evaluated by the analysis of variance (ANOVA) and Tukey post hoc test at a significance level of 0.05 using Graph Pad Prism 8.0.1 (GraphPad Software, La Jolla, CA, USA). A value of *p* < 0.05 was considered statistically significant, to determine if there was a statistical difference between the groups, and the normality and lognormality tests (Shapiro–Wilk test) were also used. All assays were performed in triplicates in two independent experiments.

## 3. Results

### 3.1. Chemical Characterization of Andiroba Oil

**Figure 1:**
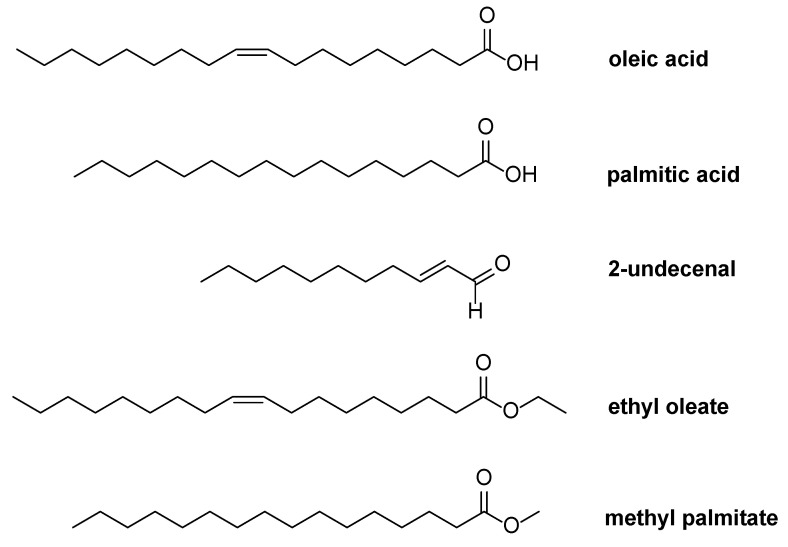
Bioactive compounds of andiroba fixed oil (*Carapa guianensis*) analyzed by gas chromatography–mass spectrometer (GC-MS).

Before starting the preparation of NeAnd, the quality and properties of andiroba oil were evaluated. The fatty acid profile analysis of andiroba oil demonstrated the presence of saturated and unsaturated fatty acids, with a predominance of oleic acid (47.62% ± 0.426) and palmitic acid (28.6% ± 0.343), and lower contents of linoleic (9.65% ± 0.145) and stearic acid (8.83% ± 0.089). Additionally, other bioactive compounds were identified in andiroba oil by gas chromatography–mass spectrometry (GC-MS), such as 2-undecenal, ethyl oleate, and methyl palmitate (Figure 1).

The andiroba oil was also evaluated in terms of its acid value, peroxide value, and free fatty acid content to assess the oil’s quality. Results showed an acid value of 5.17 ± 0.30 (mg KOH/g), a peroxide value of 1.44 ± 0.0076 meq O_2_/Kg), and free fatty acids of 2.60 ± 0.15 (%).

#### 3.1.1. Nuclear Magnetic Resonance (NMR) Analysis of Andiroba Oil

**Figure 2:**
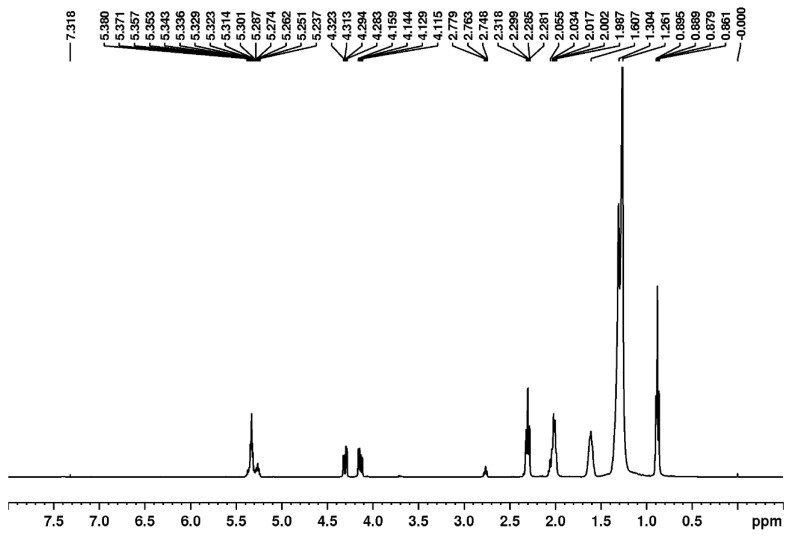
^1^H NMR spectrum of andiroba fixed oil (*Carapa guianensis*) in CDCl_3_ at 400 MHz.

**Figure 3:**
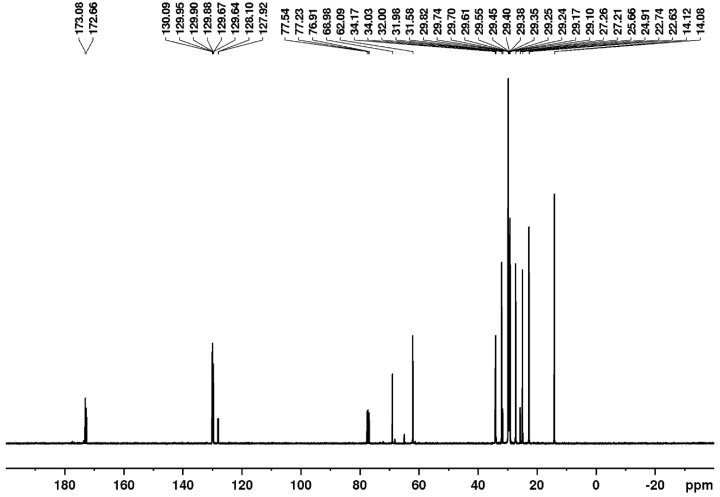
^13^C{^1^H} NMR spectrum of andiroba fixed oil (*Carapa guianensis*) in CDCl_3_ at 100 MHz.

Nuclear magnetic resonance (NMR) is a spectroscopic technique that provides the identification of primary and secondary metabolites in complex mixtures. The ^1^H and ^13^C NMR spectral profiles revealed the presence of fatty acids esterified to the glycerol moiety due to the existence of signals in the region between *δ*_H_ 4.10 to 5.50 ppm (Figure 2) and the signals at *δ*_C_ 62.09 and 68.98 ppm (Figure 3), respectively [29]. The presence of the signals at 172.66 and 173.08 ppm can be attributed to the presence of carbonyl groups in a fatty acid chain (Figure 3).

In addition, due to the presence of diagnostic chemical shifts, it was possible to assign the signals in the ^1^H NMR spectrum to some fatty acids’ chemical structural characteristic. The chemical shifts at *δ*_H_ 0.88 ppm indicates the presence of terminal methyl hydrogens of stearic acid, while the signal at *δ*_H_ 0.89 ppm was attributed to palmitic and oleic acids (Figure 2). The presence of stearic, palmitic, and oleic acid is also affirmed by the chemical signals in the region between *δ*_H_ 1.5 to 2.5 ppm, which are related to *b*-methylene hydrogens from carbonyl carbon (*δ*_H_ 1.5–1.7 ppm), allyl methylene hydrogens (*δ*_H_ 1.9–2.1 ppm), and *a*-methylene hydrogens adjacent to the carbonyl group (*δ*_H_ 2.2–2.4 ppm). In a lower intensity, it was possible to observe signals related to divinyl methylene hydrogens, characteristic of linoleic acid (*δ*_H_ 2.7–2.9 ppm) [30,31]. Regarding the ^13^C NMR spectrum, the signals at *δ*_C_ 14.08, 24.91, 29 to 31, 130.09 ppm are related to the methyl, *b*-methylene from carbonyl carbon, methylene, and sp^2^ methine carbon from oleic acid, respectively (Figure 3) [31].

#### 3.1.2. Antioxidant Activity, Total Phenolic Content, and Carotenoid Content of Andiroba Oil

As shown in Table 1, the antioxidant activity of OA was assessed using two different methods (phosphomolybdenum complex: 741.47 mg/100 g; and DPPH•: 41.28% discoloration). Total phenolic content was 338.92 mg/100 g (Table 1). A total of 8.69 μg/g of carotenoids was found in OA, including α-, β-, δ-, and γ-carotenes, as well as lycopene (Table 1).

### 3.2. Hydrodynamic Diameter, Polydispersity Index, and Zeta Potential of NeAnd

**Figure 4:**
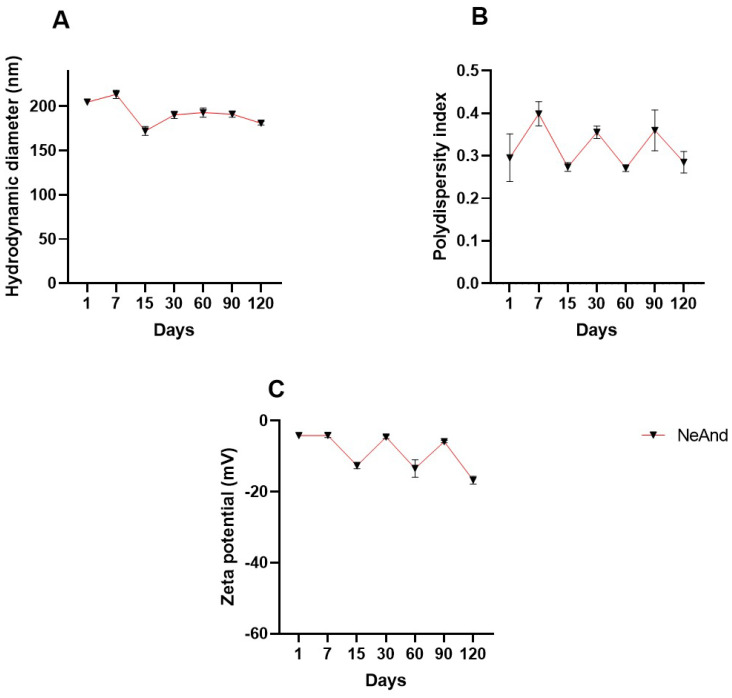
Evaluation of stability of andiroba fixed oil-based nanoemulsion (NeAnd) stored at 4 °C for 120 days. Hydrodynamic diameter (**A**); polydispersity index (**B**); and zeta potential (**C**). The values are expressed as mean ± SD.

NeAnd exhibited an average hydrodynamic diameter of 205.7 ± 3.9 nm, a polydispersity index (PdI) of 0.295 ± 0.05, and a negative zeta potential of −4.16 ± 0.414 and maintained a pH of approximately 6.5 over 120 days of storage at 4 °C (Figure 4).

#### 3.2.1. Stability Evaluation of NeAnd Under pH Stress

**Figure 5:**
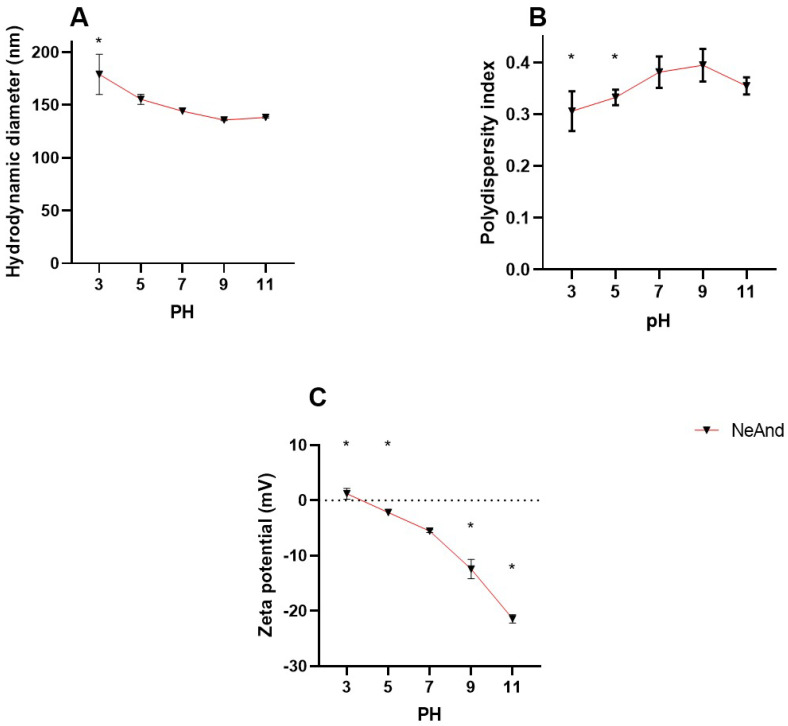
Evaluation of the stability of andiroba fixed oil-based nanoemulsion (NeAnd) under different pH conditions: hydrodynamic diameter (**A**), polydispersity index (**B**), and zeta potential (**C**). The values are expressed as mean ± SD. Two-way ANOVA: significant difference pH 7 versus other groups (*p* < 0.05, Sidak test). Asterisks indicate statistically significant differences compared to the pH 7 group.

Variations in pH values resulted in changes in NeAnd’s physicochemical characteristics related to HD and zeta potential parameters (Figure 5). When compared to nanoemulsion at pH 7, slight variations of approximately 34 nm were observed in the HD (*p* < 0.0001) under acidic conditions, with no significant differences detected under basic conditions (Figure 5A). PdI values showed slight variations (approx. 0.0836 and 0.5730, *p* < 0.0001, at pH 3 and 5, respectively) (Figure 5B). Regarding the zeta potential, values increased 6.7 mV at acidic pH and decreased approximately −15.88 mV at basic pH (*p* < 0.0001) (Figure 5C).

#### 3.2.2. Transmission Electron Microscopy (TEM) of NeAnd

**Figure 6:**
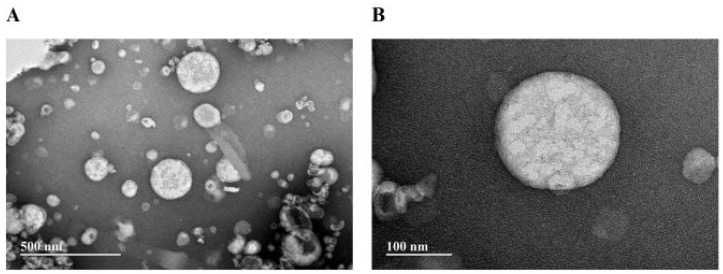
Transmission electron microscopy micrographs of andiroba fixed oil-based nanoemulsion (NeAnd). (**A**) Overview of the nanodroplets. (**B**) Magnified view of a single nanodroplet, illustrating its morphology in greater detail.

Figure 6 presents TEM images of NeAnd, in which the nanodroplets exhibit a spherical shape.

#### 3.2.3. Infrared Spectra (FTIR) of NeAnd

**Figure 7:**
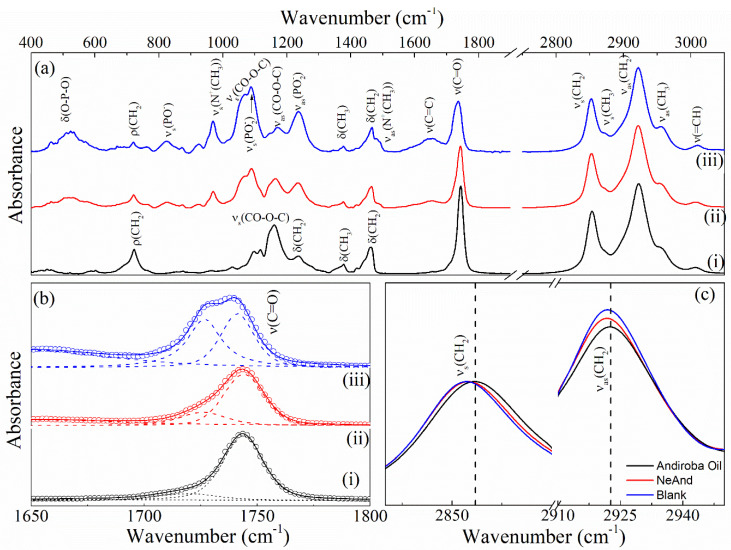
FTIR spectra of the (**a**) free andiroba fixed oil (i), blank formulation (ii), and andiroba oil-based nanoemulsion (NeAnd) (iii). Zoom of the FTIR spectra in the range 1650–1800 cm^−1^ (**b**) and the range 2840–2950 cm^−1^ (**c**). The dashed lines show the Gaussian + Gaussian + Lorentzian fit of the data.

Figure 7 shows the FTIR spectra of andiroba oil (i), andiroba oil-based nanoemulsion (NeAnd) (ii), and the blank formulation (without the oil). The FTIR spectrum of the andiroba oil (Figure 7(i)) shows typical bands related to the acyl chain of lipids, such as the stretching (=CH) of the *cis* aliphatic doublet bond at 3010 cm^−1^ and the vibrational modes of symmetric and antisymmetric stretching of (CH_2_) and (CH_3_) at 2850, 2920, 2870, and 2960 cm^−1^, respectively [32].

#### 3.2.4. Raman of NeAnd

**Figure 8:**
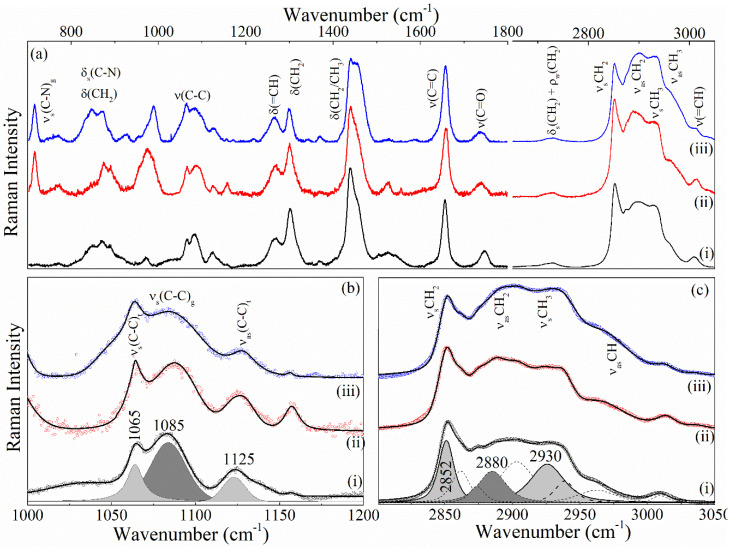
Raman spectra of the (**a**) free andiroba fixed oil (i), blank formulation (ii), and andiroba oil-based nanoemulsion (NeAnd) (iii). Zoom of the FTIR spectra in the range 1000–1200 cm^−1^ (**b**) and the range 2800–3050 cm^−1^ (**c**). The dashed lines show the Gaussian + Gaussian + Lorentzian fit of the data.

Figure 8 shows the Raman spectra of OA, blank formulation, and NeAnd. To determine the ratios between the intensities of *trans*/*gauche*, the Raman spectra were fitted using a Gaussian + Lorentzian function. The values obtained for these ratios are listed in Table 2. Note from Table 2 that the *trans*/*gauche* ratio is higher for the blank formulation and NeAnd when compared to OA. This behavior indicates that the lipid chains of NeAnd and the blank formulation are more ordered than those of free OA, which is consistent with the FTIR data.

### 3.3. Cytotoxicity of NeAnd in Human Keratinocytes In Vitro

**Figure 9:**
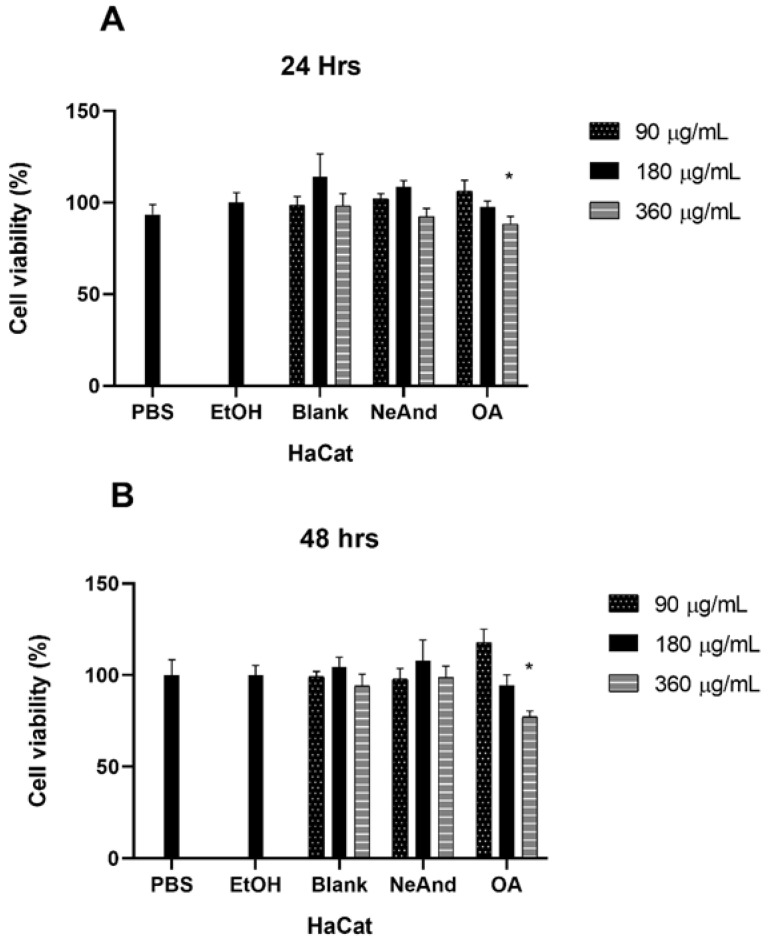
Cytotoxicity of andiroba fixed oil-based nanoemulsion (NeAnd), free andiroba oil (OA), and blank formulation (without oil) on the viability of human keratinocytes after exposure for 24 h (**A**) and 48 h (**B**). EtOH (ethanol 1%). Two-way ANOVA: significant difference among control vs. treatment groups (*p* < 0.05, Tukey post hoc test). * indicates statistically significant differences compared to the PBS group.

Cell viability analyses of human keratinocytes showed no statistically significant differences in cytotoxicity among the tested and controls at 24 and 48 h, except for the treatment of free OA at a concentration of 360 µg/mL at the 48 h time point, which resulted in an average cell viability of 77.29% (*p* < 0.05) (Figure 9A,B).

Considering that NeAnd exhibited no significant cytotoxicity in any of the concentrations evaluated above, the highest concentration (360 µg/mL) was chosen for the scratch assays.

### 3.4. Scratch Assay in Keratinocytes

**Figure 10:**
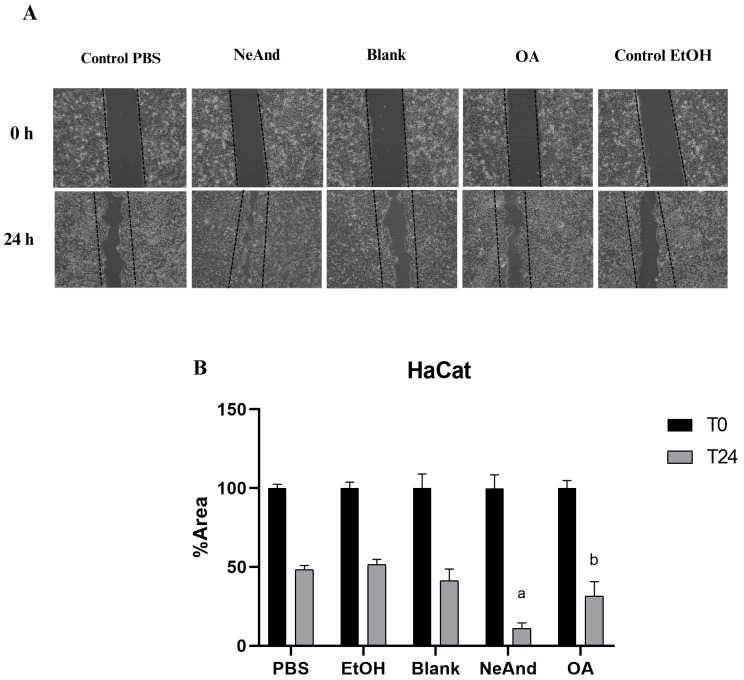
Scratch assay of keratinocytes in vitro: (**A**) simulated wound in human keratinocytes. Images were obtained before (T0) and after 24 h of incubation (T24) with PBS, NeAnd (360 µg/mL), blank (egg lecithin without oil), free andiroba fixed oil (OA-360 µg/mL), or control EtOH. Microscopy image obtained using a 10× objective lens (final magnification: 100×). (**B**) Quantification of the injury area at T0 and T24. One-way ANOVA, Tukey post hoc test. Letters indicate significant differences among groups (*p* < 0.05).

Figure 10A shows representative images of wound closure in a keratinocyte monolayer before (T0) and after 24 h (T24) of treatment with NeAnd or free OA. Figure 10B presents the percentage of the remaining wound area after treatments. The NeAnd group induced a significantly greater proportion of wound closure (89.9%) when compared to the PBS control (38%; *p* < 0.0005) and to free OA (68.6%; *p* < 0.0323).

## 4. Discussion

The present study aimed to develop and characterize a nanoemulsion based on andiroba fixed oil (NeAnd) and evaluate whether the nanoencapsulation of OA could influence its cytotoxic and wound healing properties. To achieve this, we investigated the biocompatibility of NeAnd and free OA at different concentrations, as well as their impact on keratinocyte migration in an in vitro wound model (scratch assay).

Initially, the physicochemical properties of andiroba oil were analyzed to confirm its composition. The lipid profile, including unsaturated and saturated fatty acid content, was determined, revealing the presence of oleic, palmitic, linoleic, and stearic acids in proportions similar to those reported in the literature [33]. Gas chromatography–mass spectrometry (GC-MS) further confirmed the presence of oleic and palmitic acids, along with other bioactive compounds, such as 2-undecenal (Figure 1). These findings were consistent with NMR spectral data (Figure 2 and Figure 3).

The acidity, peroxide value, and free fatty acid content of OA were also examined, revealing values lower than those previously reported in the literature [34]. These results suggest that the oil had not undergone rancidification, confirming its chemical stability. To date, and to the best of our knowledge, there are no standardized parameters established for andiroba oil. Therefore, further studies are needed to define specific physicochemical reference values for *C. guianensis* oil.

The antioxidant properties and bioactive compound content of various plant species have been strongly linked to immune system enhancement and protection against oxidative stress and disease development [35]. Since multiple mechanisms contribute to antioxidant capacity, different methods are required to assess these effects [36].

To evaluate the total antioxidant activity, we applied the phosphomolybdenum complex assay, which measures the reduction of Mo(VI) to Mo(V) in the presence of electron-donating compounds. This process occurs under acidic conditions and involves the application of high temperatures for a relatively extended period [37]. OA exhibited strong antioxidant activity (741.47 ± 23.23 mg/g) (Table 1). Notably, essential oils from *Ocimum basilicum* (basil) and *Thymus algeriensis* showed significantly lower antioxidant activities, with values of 76 mg/g and 43.2 mg/g, respectively [38].

A complementary evaluation was performed using the DPPH• free radical scavenging assay, which quantifies the ability of antioxidants to neutralize radicals. This assay relies on the discoloration of a DPPH• solution upon interaction with antioxidants such as ascorbic acid, tocopherol, phenolic compounds, and carotenoids. Compared to a control solution, OA exhibited low antioxidant activity (41.28%) using this method (Table 1). These results reflect methodological differences in antioxidant evaluation, as bioactive compounds may exhibit varying extraction efficiencies depending on the solvent and analytical technique used.

Phenolic compounds are the most abundant class of plant-derived antioxidants, characterized by aromatic rings linked to hydroxyl groups. In previous studies, the total phenolic content (TPC) of oils from different plant species [39,40,41] has ranged widely, from 108.11 mg/100 g in faveleira seed oil [42] to 900–1034 mg/100 g in *Carapa procera* oils [43]. Our findings demonstrated that the OA used in the present study contains 338.92 ± 8.55 mg/100 g of phenolic compounds, placing it within the range of moderate phenolic content [44,45]. Although andiroba oil is not a fruit, its phenolic content suggests that it may serve as a valuable source of natural antioxidants for human health applications.

Carotenoids are lipophilic pigments synthesized by various organisms, including plants, and are associated with numerous health benefits [46]. β-carotene and α-carotene serve as precursors of vitamin A, capable of being converted into retinol through dioxygenase enzyme activity [46]. Lycopene stands out as a potent and effective inhibitor of reactive oxygen species due to its conjugated double bonds and elongated structure. This bioactive compound helps to protect DNA and suppresses mutations that can lead to chronic diseases [47]. Based on the classification of carotenoid content in dietary sources, when a plant-based product contains 500–2000 μg per 100 g, it is considered a high-carotenoid source [46]. Therefore, andiroba oil in this study may be qualified as a rich source of these antioxidant bioactive compounds. It has been reported that excessive ROS generated in chronic wounds lead to oxidative stress and impair the wound healing [48]. In this context, the antioxidant activity of andiroba oil may also contribute to reducing oxidative stress and promoting wound healing. Following the chemical and antioxidant characterization of OA, the nanoemulsion formulation (NeAnd) was developed and analyzed. The hydrodynamic diameter (HD) of NeAnd fell within the expected range for phosphatidylcholine-based nanoemulsions (150–300 nm) [49,50,51]. TEM analysis showed that NeAnd nanodroplets have a spherical shape (Figure 6). It is important to highlight that, in the present work, TEM was not used to determine the size distribution of the nanodroplets, as this technique can introduce artifacts in nanoemulsion preparations. The process of capturing images of nanoemulsions with conventional TEM is complex, requiring special preparations that involve staining agents and sample evaporation. Dehydration and drying can cause significant structural changes in colloidal systems, including shrinkage, collapse, and aggregation. Furthermore, staining with heavy metal salts results in selective visualization, as only certain structures react with or are accessible to the staining agents [52].

The stability of NeAnd was assessed by monitoring the HD, zeta potential (ZP), and polydispersity index (PdI) over 120 days at 4 °C. As expected, the zeta potential remained slightly negative (~−7 mV) due to the presence of anionic emulsifiers such as egg lecithin [53]. Minor variations in HD and PdI were observed throughout storage (Figure 4). Additionally, pH analysis showed that NeAnd remained stable across different pH conditions, except at pH 3 and pH 5 (Figure 5), as expected. These variations may be attributed to the binding of cationic or anionic ions to the nanoparticle surface, which shifts the ZP to more positive or negative values, respectively [54,55,56,57]. These results suggest that NeAnd maintains adequate colloidal stability for 120 days at 4 °C, which is crucial for preserving its bioavailability in different biological systems [53,54,55].

Vibrational spectroscopy techniques like FTIR and Raman spectroscopy are valuable for investigating chemical interactions among bioactive molecules in lipid nanoemulsions [58,59,60]. Figure 7 shows the FTIR spectra of andiroba oil (OA) (i), andiroba oil-based nanoemulsion (NeAnd) (ii), and blank formulation (without the oil). Andiroba oil (*Carapa guianensis Aubl*) is constituted basically of fatty triglyceride (TG) esters with different substitution patterns, lengths, and degrees of saturation of the chains and other minor components. Its absorption spectra have characteristics common to most vegetable oil spectra, as previously reported [32,61]. The FTIR spectrum of OA shows typical bands related to the acyl chain of lipids, such as the stretching (=CH) of the *cis* aliphatic doublet bond at 3010 cm^−1^ and the vibrational modes of symmetric and antisymmetric stretching of (CH_2_) and (CH_3_) at 2850, 2920, 2870, and 2960 cm^−1^, respectively [32]. Additionally, other vibrations associated with (CH_2_) and (CH_3_) bonds are observed at 1460 cm^−1^ (CH_2_ scissoring), 1376 cm^−1^ (CH_3_ symmetric deformation (umbrella)), 1234 cm^−1^ (CH_2_ twisting), and 720 cm^−1^ (ρ(CH_2_)). The intense band at 1160 cm^−1^ is likely due to stretching vibrations of the C-CO-C) bonds of the carbonyl or ketone groups [62]. Additionally, a band assigned with the ν(C=O) stretching steres and a weak band associated with the ν(C=C)_cis_ vibrations of unsaturated bonds are observed at 1745 and 1650 cm^−1^, respectively [63].

Comparisons among the FTIR spectra of the nanoemulsion, blank formulation, and free OA reveal that the NeAnd spectrum closely resembles the IR absorption spectrum of the blank formulation, displaying absorption bands characteristic of the polar head of lecithin. This similarity is particularly pronounced in the spectral region of 800–1350 cm^−1^, where vibrational modes associated with symmetric (~1090 cm^−1^) and antisymmetric (~1235 cm^−1^) stretching modes of the $PO_{2}^{-}$ groups, partially overlap with the symmetric stretching modes of the CO-O-C bond (~1065 cm^−1^). Furthermore, symmetric and antisymmetric stretching bands of $N^{+}\left(CH_{3}\right)$ bonds are found at 970 and 1480 cm^−1^, respectively.

Note from Figure 7b that the spectral profile of the ν(C=O) band can be deconvolved into two components using the Gaussian + Lorentzian function. As previously reported, these bands can be attributed to populations of ‘free’ (~1743 cm^−1^) and bonded’ (~1725 cm^−1^) ester carbonyl groups via hydrogen bonds. It is noteworthy that the intensity of the component *ν*(*C* = *O*)*livre* decreases with the introduced andiroba oil (see Figure 7b(iii) and (ii)). This result suggests that the lipid chains comprising the membrane of the blank formulation are more ordered than those of NeAnd, which in turn are more ordered than those of free andiroba oil. This hypothesis is supported by the behavior of the vibrational energies of the modes and, which decrease by approximately 1.5 cm^−1^, in the spectra of the structured samples when compared to the free andiroba oil.

Figure 8 shows the Raman spectra of the free andiroba oil (i), NeAnd, (ii), and blank formulations (iii). Except for the peak at 720 cm^−1^ (ν(CN)), the Raman spectra of NeAnd and blank formulations closely resemble the Raman spectrum of free andiroba oil. The Raman spectra of phospholipids present various peaks, which can be classified into three regions: (i) the hydrophobic chain consisting of C-H stretching modes (symmetric and asymmetric stretching at 2852 and 2880 cm^−1^ and 2930 and 2960 cm^−1^, respectively), deformation (~1450 cm^−1^), twisting (1300 cm^−1^), and C-C stretching (1000–1200 cm^−1^) modes, (ii) the interfacial regions containing C=O stretching, and (iii) polar headgroup regions comprising a band of C-N stretching at 720 cm^−1^ [64].

The relative intensities of Raman active modes arising from the hydrophobic chains of hydrocarbons have been utilized to identify TG polymorphs and to probe the conformation, environment, and dynamics of hydrocarbon chains in TGs. The band $\nu_{s}\left(CH_{3}\right)$ at 2930 cm^−1^ and the band ν(C-C) at 1085 cm^−1^ can be used as a general measure of *gauche* content [65]. On the other hand, the bands $\nu_{s}\left(CH_{3}\right)$ (2930 cm^−1^), $\nu_{as}\left(C-C\right)\;$(1065 cm^−1^) and $\nu_{s}\left(C-C\right)\;$(1125 cm^−1^) are associated with ordered *all-trans* conformation. Hence, the *trans/gauche* ($I_{t}/I_{g})$ ratio (($I_{1065}/I_{1085}$, $I_{1125}/I_{1085}$ and $I_{2880}/I_{2930}$) can be employed to ascertain the relative content of gauche rotamers, serving as a measure for intra- and intermolecular disorders. Furthermore, the ratio between $I_{2880}/I_{2850}$ assesses the strength of the lateral chain–chain interaction within the lipid layer [66].

In order to calculate the ratios between the intensities $I_{1065}/I_{1085}$, $I_{1125}/I_{1085}$, $I_{2880}/I_{2850}$, and $I_{2880}/I_{2930}$, the Raman spectra were fitted using the Gaussian + Lorentzian function. The values obtained for these ratios are listed in Table 2. Note from Table 2 that the $I_{t}/I_{g}$ ratio is higher for blank formulation and NeAnd compared to andiroba oil. This suggests that the lipid chains of the NeAnd and blank formulation are more ordered than those of free andiroba oil, which is in agreement with the FTIR data.

Finally, biocompatibility assessments were performed using human keratinocytes, given their critical role in wound healing. The results showed that a 24- and 48 h incubations of NeAnd and OA in keratinocytes did not result in significant cytotoxicity at the evaluated concentrations, except for OA at 360 ug/mL after 48 h, which reduced cell viability by approximately 25%. These results indicate the potential biocompatibility of NeAnd with the cells involved in the wound healing process. Several studies have examined the use of andiroba oil and its cytotoxicity. For instance, Milhomem-Paixão et al. 2017 [67] investigated an andiroba-based nanoemulsion and arrived at conclusions similar to those obtained in this study. They emphasized that OA exhibited higher toxicity than nanoformulations, observing a direct correlation between the concentration of oil and its cytotoxicity. A more recent study conducted by Porfírio-Dias et al. 2020 [68] further confirmed that higher OA concentrations and longer exposure times to the free oil led to increased cytotoxicity.

The in vitro scratch assay demonstrated that NeAnd (360 µg/mL) significantly improved keratinocyte migration and wound closure compared to free OA. After 24 h, untreated cells exhibited 38% closure, while cells treated with NeAnd and OA reached 88.9% and 68.6%, respectively (Figure 10). It is well established in the literature that oil extracted from andiroba seeds exhibits anti-inflammatory and wound healing properties. When topically applied to wounds, OA can help reduce inflammation, alleviate pain, and promote tissue regeneration [13,69,70]. These therapeutic effects are associated with the fatty acids present in this oil, such as linoleic acid and oleic acid, which have demonstrated a positive role in wound healing, due to their ability to modulate inflammation and enhance the in vivo reparative response [71]. These fatty acids have also proven effective in increasing neutrophil presence in the wound and reducing necrotic tissue thickness [72]. These compounds were confirmed in the andiroba oil that was used in this study, emphasizing that the nanostructured version alters its bioavailability and enhances cell migration in keratinocytes compared to the free OA.

It is well understood that the wound healing process is highly complex and involves additional cell types such as fibroblasts and macrophages, as well as multiple signaling pathways [1,2,3]. This can be addressed in future studies by advancing to in vivo models and, subsequently, to clinical trials to confirm the efficacy of this formulation in promoting wound healing. Our research group is aware of this need and is currently conducting wound healing studies in in vivo models to further investigate NeAnd effects and unravel the potential of this promising nanophytomedicine.

## 5. Conclusions

In the present work, we successfully developed and characterized a nanoemulsion based on andiroba fixed oil (NeAnd), emphasizing the key steps involved in its production—from the identification of the main compounds and characteristics of the natural product (OA) to the physicochemical properties, stability, and morphology of the obtained nanodroplets. Techniques including dynamic light scattering (DLS), electrophoretic light scattering (ELS), transmission electron microscopy (TEM), FTIR, and Raman were used to characterize the nanodroplets. NeAnd was spherical and remained stable for over 120 days at 4 °C, with a hydrodynamic diameter of approximately 205 nm. Furthermore, our nanotechnological approach improved andiroba oil’s effect on wound healing in vitro, stimulating keratinocyte migration and accelerating wound closure. It is noteworthy to highlight that NeAnd was biocompatible, showing no significant cytotoxicity at any of the time points or concentrations evaluated. To the best of our knowledge, there are no published data on nanoformulations that have tested the wound healing effect of nanostructured andiroba oil in vitro. These promising data point out NeAnd as a potential nanophytomedicine for clinical application in wound healing and tissue regeneration treatments. It is well known that in vitro studies are valuable approaches for investigating specific cellular effects and mechanisms. Considering the promising results obtained herein and the fact that wound healing involves angiogenesis and various cell types beyond keratinocytes, such as fibroblasts and immune cells, investigations using in vivo models are currently underway.

## Figures and Tables

**Table 1:** Antioxidant activity (phosphomolybdenum complex and DPPH•), total phenolic compounds (TPCs), and carotenoids detected in andiroba fixed oil.

Parameter	Value Obtained	
Phosphomolybdenum complex	741.47 ± 23.23	mg/100 g
DPPH•	41.28 ± 1.45	discoloration %
TPC	338.92 ± 8.55	mg/100 g
α-Carotene	1.88 ± 0.28	μg/g
β-Carotene	1.86 ± 0.32	μg/g
δ-Carotene	1.76 ± 0.51	μg/g
γ-Carotene	2.05 ± 0.42	μg/g
Lycopene	1.15 ± 0.50	μg/g
Total carotenoids	8.69 ± 1.42	μg/g

**Table 2:** Calculated $I_{t}/I_{g}$ ratio (from Raman spectra data) for free andiroba fixed oil, blank formulation, and andiroba oil-based nanoemulsion (NeAnd).

Samples	*I*_1065_/*I*_1085_	*I*_1125_/*I*_1085_	*I*_2880_/*I*_2850_	*I*_2880_/*I*_2930_
Andiroba oil	0.63	0.40	0.38	0.81
Blank	1.12	0.60	0.50	1.06
NeAnd	1.02	0.59	0.52	1.02

## Data Availability

Dataset available on request from the authors.

## References

[1] Wilkinson H.N., Hardman M.J. (2020). Wound healing: Cellular mechanisms and pathological outcomes. Open Biol..

[2] Gurtner G.C., Werner S., Barrandon Y., Longaker M.T. (2008). Wound repair and regeneration. Nature.

[3] Peña O.A., Martin P. (2024). Cellular and molecular mechanisms of skin wound healing. Nat. Rev. Mol. Cell Biol..

[4] Dias K.K.B., Cardoso A.L., Costa K.M.P., de Souza A.A.F., Passos M.F., Costa C.E.F., Rocha-Filho G.N., Andrade E.H.A., Luque R., Nascimento L.A.S. (2023). Biological activities from andiroba (*Carapa guianensis* Aublet.) and its biotechnological applications: A systematic review. Arab. J. Chem..

[5] Bayda S., Adeel M., Tuccinardi T., Cordani M., Rizzolio F. (2019). The History of Nanoscience and Nanotechnology: From Chemical–Physical Applications to Nanomedicine. Molecules.

[6] Jiang T., Li Q., Qiu J., Du S., Xu X., Wu Z., Yang X., Chen Z., Chen T. (2022). Nanobiotechnology: Applications in Chronic Wound Healing. Int. J. Nanomed..

[7] Chopra H., Mohanta Y.K., Mahanta S., Mohanta T.K., Singh I., Avula S.K., Mallick S.P., Rabaan A.A., AlSaihati H., Alsayyah A. (2023). Recent updates in nanotechnological advances for wound healing: A narrative review. Nanotechnol. Rev..

[8] Lucca L.G., Matos S.P., Kreutz T., Teixeira H.F., Veiga V.F., Araújo B.V., Limberger R.P., Koester L.S. (2018). Anti-inflammatory Effect from a Hydrogel Containing Nanoemulsified *Copaiba oil* (*Copaifera multijuga* Hayne). AAPS PharmSciTech.

[9] Blanco-Fernandez B., Castaño O., Mateos-Timoneda M.Á., Engel E., Pérez-Amodio S. (2020). Nanotechnology approaches in chronic wound healing. Adv. Wound Care.

[10] Bajerski L., Michels L.R., Colomé L.M., Bender E.A., Freddo R.J., Bruxel F., Haas S.E. (2016). The use of Brazilian vegetable oils in nanoemulsions: An update on preparation and biological applications. Braz. J. Pharm. Sci..

[11] Giácomo R.G., Pereira M.G.G., Silva C.F., Gaia-Gomes J.H. (2017). Litter and carbon deposition in secondary forest, Sabia and Andiroba plantations. Floresta.

[12] Oliveira R.S., Fernandes M.M.P., Mesquita M.N., Cruz A.C.L., Pelizzari C., Neves E.C., Peruquetti R.C., Berón M.M., Viott A.M., Souza S.F. (2021). Therapeutic laser with or without andiroba oil in treating cutaneous wounds by second intention in Wistar rats. Acta Vet. Bras..

[13] Chia C.Y., Medeiros A.D., Corraes A.M.S., Manso J.E.F., Silva C.S.C., Takiya C.M., Vanz R.L. (2018). Healing effect of andiroba-based emulsion in cutaneous wound healing via modulation of inflammation and transforming growth factor beta 3. Acta Cir. Bras..

[14] Souza B.A.A., Braga L.A., Lopes R.L.O., Ribeiro-Junior R.F.G., Nascimento L.N.S., Cavalcante L.C.C., Monteiro A.M., Couteiro R.P., Yasojima E.Y., Hamoy M. (2017). Effects of Andiroba oil (*Carapa guianensis*) on wound healing in alloxan-diabetic rats. Int. Arch. Med..

[15] Silva C.E.S., Santos O.J., Ribas-Filho J.M., Tabushi F.I., Kume M.H., Jukonis L.B., Cella I.F. (2023). Effect of Carapa guianensis Aublet (Andiroba) and Orbignya phalerata (Babassu) in colonic healing in rats. Ver. Cor. Bras. Cir..

[16] Araújo A.L., Teixeira F.A., Lacerda T.F., Flecher M.A., Souza V.R.C., Coelho C.S. (2017). Effects of topical application of pure and ozonized andiroba oil on experimentally induced wounds in horses. Braz. J. Vet. Res. Anim. Sci..

[17] Ombredane A.S., Araújo V.H.S., Borges C.O., Costa P.L., Landim M.G., Pinheiro A.C., Szlachetka I.O., Benedito L.E.C., Espindola L.S., Dias D.J.S. (2020). Nanoemulsion-based systems as a promising approach for enhancing the antitumoral activity of pequi oil (*Caryocar brasilense* Cambess.) in breast cancer cells. J. Drug. Deliv. Sci. Technol..

[18] Allaw M., Manconi M., Caboni P., Bacchetta G., Escribano-Ferrer E., Peris J.E., Nacher A., Diez-Sales O., Manca M.L. (2021). Formulation of liposomes loading lentisk oil to ameliorate topical delivery, attenuate oxidative stress damage and improve cell migration in scratch assay. Biomed. Pharmacother..

[19] Morganti P. (2015). Bionanotechnology & Bioeconomy for a Greener Development. J. Appl. Cosmetol..

[20] Nasser K., Petru-Cristian N. (2023). View of Nanotechnology: A Tiny Solution for the Big Challenges in Agriculture. Int. J. Res. Adv. Agric. Sci..

[21] AOCS (2004). Official Methods and Recommended Practices of the American Oil Chemists’ Society.

[22] Prieto P., Pineda M., Aguilar M. (1999). Spectrophotometric Quantitation of Antioxidant Capacity through the Formation of a Phosphomolybdenum Complex: Specific Application to the Determination of Vitamin E. Anal. Biochem..

[23] Rufino M.S.M., Alves R.E., Brito E.S., Pérez-Jiménez J., Saura-Calixto F., Mancini-Filho J. (2010). Bioactive compounds and antioxidant capacities of 18 non-traditional tropical fruits from Brazil. Food Chem..

[24] Medina M.B. (2011). Determination of the total phenolics in juices and superfruits by a novel chemical method. J. Funct. Foods..

[25] Rodriguez-Amaya D.B. (2001). A Guide to Carotenoid Analysis in Foods.

[26] Mosmann T. (1983). Rapid colorimetric assay for cellular growth and survival: Application to proliferation and cytotoxicity assays. J. Immunol. Methods.

[27] Liakopoulou A., Mourelatou E., Hatziantoniou S. (2021). Exploitation of traditional healing properties, using the nanotechnology’s advantages: The case of curcumin. Toxicol. Rep..

[28] Suarez-Arnedo A., Figueiroa F.T., Clavijo C., Arbeláez P., Cruz J.C. (2020). An image J plugin for the high throughput image analysis of in vitro scratch wound healing assays. PLoS ONE.

[29] Santos O.N.A., Folegatti M.V., Dutra L.M., Andrade I.P.S., Fanaya E.D., Lena B.P., Barison A., Santos A.D.C. (2017). Tracking lipid profiles of *Jatropha curcas* L. seeds under different pruning types and water managements by low-field and HR-MAS NMR spectroscopy. Ind. Crops Prod..

[30] Miyake Y., Yokomizo K., Matsuzaki N. (1998). Determination of unsaturated fatty acid composition by high-resolution nuclear magnetic resonance spectroscopy. JAOCS.

[31] Di Pietro M.E., Mannu A., Mele A. (2020). NMR Determination of Free Fatty Acids in Vegetable Oils. Processes.

[32] Santana F.B., Mazivila S.J., Gontijo L.C., Neto W.B., Poppi R.J. (2018). Rapid Discrimination Between Authentic and Adulterated Andiroba Oil Using FTIR-HATR Spectroscopy and Random Forest. Food Anal. Methods.

[33] Lozano-Garzón K., Orduz-Díaz L.L., Guerrero-Perilla C., Quintero-Mendoza W., Carrillo M.P., Cardona-Jaramillo J.E.C. (2023). Comprehensive Characterization of Oils and Fats of Six Species from the Colombian Amazon Region with Industrial Potential. Biomolecules.

[34] Ferreira A.M., Sena I.S., Magalhães K.F., Oliveira S.L., Ferreira I.M., Porto A.L.M. (2018). Amazon Oils from Andiroba (*Carapa* sp.) and Babassu (*Orbignya* sp.) for Preparation Biodiesel by Enzymatic Catalysis. Curr. Biotechnol..

[35] Viera W., Shinohara T., Samaniego I., Sanada A., Terada N., Ron L., Suárez-Tapia A., Koshio K. (2022). Phytochemical Composition and Antioxidant Activity of Passiflora spp. Germplasm Grown in Ecuador. Plants.

[36] Barros H.E.A., Natarelli C.V.L., Tavares I.M.C., Oliveira A.L.M., Araújo A.B.S., Pereira J., Carvalho E.E.N., Vilas-Boas E.V.B., Franco M. (2020). Nutritional Clustering of Cookies Developed with Cocoa Shell, Soy, and Green Banana Flours Using Exploratory Methods. Food Bioproc. Technol..

[37] Mohamed A.S., Al-Bar O.A.M., Al-Najada A.R. (2017). Chemical modification of curcumin: Solubility and antioxidant capacity. Int. J. Food Prop..

[38] Rezzoug M., Bakchiche B., Gherib A., Ascrizzi R., Flamini G., Kilinçarslan O., Mammadov R., Bardaweel S.K. (2019). Chemical composition and bioactivity of essential oils and Ethanolic extracts of *Ocimum basilicum* L. and Thymus algeriensis Boiss. & Reut. from the Algerian Saharan Atlas. BMC Complement. Altern. Med..

[39] Kala N.S., Ramasubbu R. (2021). Chemical composition, antimicrobial and antioxidant properties of essential oils of *Trichopus zeylanicus* ssp. travancoricus. Indian J. Nat. Prod. Resour..

[40] da Costa C.A.R., Machado G.G.L., Rodrigues L.J., Barros H.E.A., Natarelli C.V.L., Vilas-Boas E.V.B. (2023). Phenolic compounds profile and antioxidant activity of purple passion fruit’s pulp, peel and seed at different maturation stages. Sci. Hortic..

[41] John S., Monica S.J., Priyadarshini S., Sivaraj C., Arumugam P. (2017). Antioxidant and Antibacterial Activities of *Beta vulgaris* L. Peel Extracts. Int. J. Pharm. Sci. Res..

[42] Ribeiro P.P.C., Damasceno K.S.F.S.C., Veras B.O., Oliveira J.R.S., Lima V.L.M., Assis C.R.D., Silva M.V., Júnior F.C.S., Assis C.F., Padilha C.E.A. (2021). Chemical and biological activities of faveleira (*Cnidoscolus quercifolius* Pohl) seed oil for potential health applications. Food Chem..

[43] Seck I., Hosu A., Cimpoiu C., Ndoye S.F., Ba L.A., Sall C., Seck M. (2021). Phytochemicals content, screening and antioxidant/pro-oxidant activities of *Carapa procera* (barks) (Meliaceae). S. Afr. J. Bot..

[44] Araujo-Lima C.F., Fernandes A.S., Gomes E.M., Oliveira L.L., Macedo A.F., Antoniassi R., Wilhelm A.E., Aiub C.A.F., Felzenszwalb I. (2018). Antioxidant activity and genotoxic assessment of crabwood (andiroba, *Carapa guianensis* Aublet) seed oils. Oxidative Med. Cell Longev..

[45] Vasco C., Ruales J., Kamal-Eldin A. (2008). Total phenolic compounds and antioxidant capacities of major fruits from Ecuador. Food Chem..

[46] Elvira-Torales L.I., García-Alonso J., Periago-Castón M.J. (2019). Nutritional Importance of Carotenoids and Their Effect on Liver Health: A Review. Antioxidants.

[47] Miller A.P., Coronel J., Amengual J. (2020). The role of β-carotene and vitamin A in atherogenesis: Evidences from preclinical and clinical studies. Biochim. Biophys. Acta Mol. Cell Biol. Lipids.

[48] Fadilah N.I.M., Phang S.J., Kamaruzaman N., Salleh A., Zawani M., Sanyal A., Maarof M., Fauzi M.B. (2023). Antioxidant Biomaterials in Cutaneous Wound Healing and Tissue Regeneration: A Critical Review. Antioxidants.

[49] Klang V., Valenta C. (2011). Lecithin-based nanoemulsions. J. Drug Deliv. Sci. Technol..

[50] Fuentes K., Matamala C., Martínez N., Zúñiga R.N., Troncoso E. (2021). Comparative Study of Physicochemical Properties of Nanoemulsions Fabricated with Natural and Synthetic Surfactants. Processes.

[51] Choi S.J., McClements D.J. (2020). Nanoemulsions as delivery systems for lipophilic nutraceuticals: Strategies for improving their formulation, stability, functionality and bioavailability. Food Sci. Biotechnol..

[52] Klang V., Matsko N.B., Valenta C., Hofer F. (2012). Electron microscopy of nanoemulsions: An essential tool for characterisation and stability assessment. Micron.

[53] Chanamai R., Horn G., McClements D.J. (2002). Influence of oil polarity on droplet growth in oil-in-water emulsions stabilized by a weakly adsorbing biopolymer or a nonionic surfactant. J. Colloid Interface Sci..

[54] Avramescu M.L., Rasmussen P.E., Chénier M., Gardner H.D. (2016). Influence of pH, particle size and crystal form on dissolution behaviour of engineered nanomaterials. Environ. Sci. Pollut. Res..

[55] Li Y., Wu H., Yang X., Jia M., Li Y., Huang Y., Lin J., Wu S., Hou Z. (2014). Mitomycin C-soybean phosphatidylcholine complex-loaded self-assembled PEG-Lipid-PLA hybrid nanoparticles for targeted drug delivery and dual-controlled drug release. Mol. Pharm..

[56] Ahangar L.E., Movassaghi K., Yaghoobi F. (2022). The pH Role in Nanotechnology, Electrochemistry, and Nano-Drug Delivery. Iran. J. Chem. Chem. Eng..

[57] Bhattacharjee S. (2016). DLS and zeta potential–what they are and what they are not?. J. Control. Release.

[58] Ferreira B.S., Almeida C.G., Hyaric M.L., Oliveira V.E., Edwards H.G.M., Oliveira L.F.C. (2013). Raman spectroscopic investigation of carotenoids in oils from Amazonian products. Spectrosc. Lett..

[59] Akita C., Kawaguchi T., Kaneko F. (2006). Structural Study on Polymorphism of Cis-Unsaturated Triacylglycerol: Triolein. J. Phys. Chem. B.

[60] Lewis R.N.A.H., McElhaney R.N. (2013). Membrane lipid phase transitions and phase organization studied by Fourier transform infrared spectroscopy. Biochim. Biophys. Acta.

[61] Silva D.F., Lima K.T., Bastos G.N., Oliveira J.A.R., Nascimento L.A.S., Costa C.E.F., Filho G.N.R., Concha V.O.C., Passos M.F. (2021). PCL/Andiroba Oil (*Carapa guianensis* Aubl.) Hybrid Film for Wound Healing Applications. Polymers.

[62] Lewis R.N.A.H., McElhaney R.N. (2007). Fourier Transform Infrared Spectroscopy in the Study of Lipid Phase Transitions in Model and Biological Membranes. Methods Mol. Biol..

[63] Rohman A., Man Y.B.C. (2011). The use of Fourier transform mid infrared (FT-MIR) spectroscopy for detection and quantification of adulteration in virgin coconut oil. Food Chem..

[64] Tantipolphan R., Rades T., Strachan C.J., Gordon K.C., Medlicott N.J. (2006). Analysis of lecithin–cholesterol mixtures using Raman spectroscopy. J. Pharm. Biomed. Anal..

[65] Silva E., Rousseau D. (2008). Molecular order and thermodynamics of the solid–liquid transition in triglycerides via Raman spectroscopy. Phys. Chem. Chem. Phys..

[66] Csiszár A., Koglin E., Meier R.J., Klumpp E. (2006). The phase transition behavior of 1,2-dipalmitoyl-sn-glycero-3-phosphocholine (DPPC) model membrane influenced by 2,4-dichlorophenol—An FT-Raman Spectroscopy Study. Chem. Phys. Lipids.

[67] Milhomem-Paixão S.S.R., Fascineli M.L., Muehlmann L.A., Melo K.M., Salgado H.L.C., Joanitti G.A., Pieczarka J.C., Azevedo R.B., Santos A.S., Grisolia C.K. (2017). Andiroba Oil (*Carapa guianensis* Aublet) Nanoemulsions: Development and Assessment of Cytotoxicity, Genotoxicity, and Hematotoxicity. J. Nanomater..

[68] Porfírio-Dias C.L., Melo K.M., Bastos C.E.M.C., Ferreira T.A.A., Azevedo L.F.C., Salgado H.L., Santos A.S., Rissino J.D., Nagamachi C.Y., Pieczarka J.C. (2020). Andiroba oil (*Carapa guianensis* Aubl.) shows cytotoxicity but no mutagenicity in the ACPP02 gastric cancer cell line. J. Appl. Toxicol..

[69] Fonseca A.S.A.D., Monteiro I.S., Santos C.R., Carneiro M.L.B., Morais S.S., Araújo P.L., Santana T.F., Joanitti G.A. (2024). Effects of andiroba oil (*Carapa guianensis* Aublet) on the immune system in inflammation and wound healing: A scoping review. J. Ethnopharmacol..

[70] Tuji F.M., Figueiredo P.B.A., Cavalcante G.H.S., Burbano R.M.R., Chauhan D.N., Singh P.R., Chauhan N.S., Shah K. (2023). Evaluation of The Anti-Inflammatory Action of Andiroba Oil—*Carapa guianensis* aubl (Meliceae) in Oral Mucositis. Pharmacological Studies in Natural Oral Care.

[71] Cardoso C.R.B., Souza M.A., Ferro E.A.V., Favoreto S., Pena J.D.O. (2004). Influence of topical administration of n-3 and n-6 essential and n-9 nonessential fatty acids on the healing of cutaneous wounds. Wound Repair Regen..

[72] Pereira L.M., Hatanaka E., Martins E.F., Oliveira F., Liberti E.A., Farsky S.H., Curi R., Pithon-Curi T.C. (2008). Effect of oleic and linoleic acids on the inflammatory phase of wound healing in rats. Cell Biochem. Funct..

